# Species- and Processing-Dependent Variability of Ascorbic Acid in Fruits of 14 *Rosa* Species and its Redox Behavior toward Iron and Copper Ions

**DOI:** 10.1021/acsomega.6c00617

**Published:** 2026-05-22

**Authors:** Ain Raal, Andres Meos, Inga Ainsaar, Olga Movtšanjuk, Kreete-Lisett Remmelgas, Oleh Koshovyi, Zuzana Lomozová, Patrícia Harčárová, Přemysl Mladěnka

**Affiliations:** † Institute of Pharmacy, Faculty of Pharmacy, 37546 University of Tartu, Tartu 50411, Estonia; ‡ Department of Pharmacognosy and Pharmaceutical Botany, 37740 Charles University, Faculty of Pharmacy in Hradec Králové, Hradec Králové 50005, Czechia; § Department of Pharmacology and Toxicology, Charles University, Faculty of Pharmacy in Hradec Králové, Hradec Králové 50005, Czechia

## Abstract

Ascorbic acid (AA) is a key phytochemical marker of rosehip (*Rosa* spp.) fruits, but its functional behavior in complex plant matrices remains unclear. Here, we analyzed AA variability in fruits of 14 *Rosa* spp. (0.26–5.57% dry weight) and evaluated the effects of processing and redox activity toward iron and copper. AA content varied markedly among species and was strongly influenced by postharvest conditions, with short-term drying at 60–80 °C preserving AA most effectively, whereas prolonged drying caused degradation and freezing (−18 °C, 1 year) had no significant effect. Despite large differences in AA content, copper reduction was observed for all samples and showed no correlation with AA concentration. In contrast, iron reduction was weaker, restricted to acidic conditions (pH 4.5–5.5), and correlated with AA content at pH 4.5 after exclusion of an outlier species (*R* ^2^ = 0.72, *p* = 0.008) but not at pH 5.5. A substantial proportion of commercial samples contained little or no detectable AA, indicating high variability in product quality. These findings demonstrate that the redox behavior of rosehip fruits is governed by phytochemical matrix effects rather than AA concentration alone and highlight the importance of species selection and processing conditions in determining AA content.

## Graphical abstract

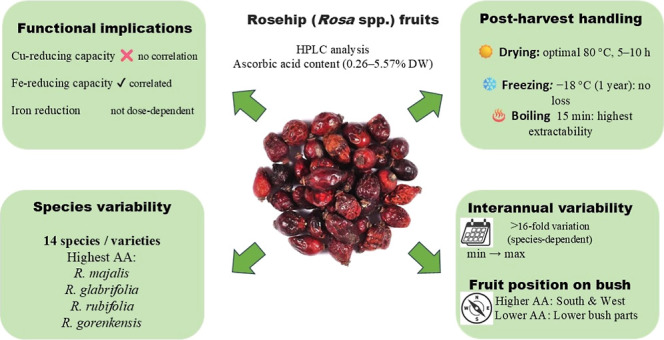

## 1 Introduction

Rosehips, the fruits of *Rosa* species (*Rosaceae*), are widely recognized for their high content of bioactive compounds and have long been used as food or in phytotherapy, in particular in traditional medicine. Beyond flavonoids,^1^ ascorbic acid (AA, vitamin C) is considered one of the most important quality-related markers of rosehip fruits due to its nutritional value and antioxidant properties.^2–5^ In fact, rosehips have repeatedly been identified as exceptionally rich sources of AA among wild fruits, with several studies highlighting their outstanding vitamin C levels in comparison with other temperate plant species.^6^ However, reported AA concentrations in rosehips vary remarkably across studies, reflecting differences among species, genotypes, and growing conditions.^7,8^

Previous studies have demonstrated that AA levels in *Rosa* fruits are influenced by species identity, genetic background, climatic conditions, harvest time, and postharvest handling.^7–9^ Interannual variability has been observed even within the same species and collection sites, suggesting that environmental factors and year-to-year climatic variation play significant roles in chemical fruit composition.^9^ In addition, processing conditions such as drying temperature and storage are known to affect vitamin C retention, often resulting in pronounced degradation under unfavorable conditions.^4,10,11^ Although antioxidant and metal-reducing properties of pure AA are likely well concentration-dependent, the effect of the rosehip fruit on metals is dependent on the combined presence of AA and other phytochemical constituents and therefore cannot be reliably predicted but must be investigated experimentally.

Iron and copper were selected as model redox-active metals because both are physiologically relevant but differ in their reduction chemistries. Fe­(III) reduction is associated with iron bioavailability, whereas Cu­(II) reduction provides a complementary readout of matrix-dependent redox activity. Assessing both metals, therefore, enables evaluation of whether rosehip redox behavior reflects AA content alone or broader phytochemical matrix effects.^12^ Rosehip fruits also contain numerous other phytochemicals, such as flavonoids, tannins, and phenolic acids, which may contribute to metal reduction or modulate the activity of AA. Consequently, the redox behavior of rosehip extracts likely reflects combined matrix effects rather than a single compound.^1,4^

Despite extensive data on AA content, the role of the rosehip phytochemical matrix in metal-ion reduction remains unclear. In particular, it is not known whether iron and copper reduction in rosehip extracts is solely governed by AA concentration or is modulated by co-occurring phytochemicals, and comparative data for both metals under physiologically relevant conditions are lacking.

We hypothesize that metal reduction in rosehip extracts is governed by phytochemical matrix effects rather than AA concentration alone, resulting in differential behavior toward iron and copper. Therefore, the present study integrates quantitative determination of AA in 14 *Rosa* species and varieties selected to capture species diversity and expected variability in AA content and postharvest conditions with copper- and iron-reduction assays under physiologically relevant pH values. By linking compositional variability with functional redox behavior, this work provides a phytochemically oriented assessment of rosehip fruits that goes beyond concentration-based evaluation of vitamin C. We have previously successfully used the HPLC method for AA determination in the analysis of various plant raw materials.^13^

## 2 Material and Methods

The plant species and varieties collected were identified by Inga Ainsaar and Ain Raal based on a special Estonian plant identifier.^14–16^ All fruits were collected at full ripeness based on color and consistency. The voucher specimen is available at the Department of Pharmacy, University of Tartu, Estonia (No Rosaceae/Rosa1-14).

### 2.1 Differences in Ascorbic Acid Content in Rosehips from Different *Rosa* spp. and the Impact of Season

The fruits of 14 rosehip species or varieties were collected in 2015 and 2023, whenever possible from the same bush (Table 1). The collected fruits were immediately transferred to a freezer at −18 °C and dried at 80 °C within 5 h. The extraction of AA^17^ and HPLC analysis continued as described in Supporting Information as Methods S1a,b and S2. The content of AA was determined by a slightly modified European Pharmacopoeia HPLC method for the quantification of related substances in AA.^18^ The same method has been successfully used in our previous studies to analyze the AA content in medical plants,^19,20^ berries,^21^ food supplements,^22^ and food plants.^13^ The detection limit of the method used is 0.3 μg/mL, and the quantification limit is 1.1 μg/mL.

**Table 1:** Content of ascorbic acid (%) in Different *Rosa* spp. Fruits Collected in 2023 and 2015 from Nature^a^

sample	Rosa spp	place of origin		content in fruits, % (dw)	
		2023	2015	2023	2015
1	Rosa pimpinellifolia	Rapla town, Rapla county	0.26 ± 0.01	0.36 ± 0.02	
2	Rosa majalis	Suigu village, Are municipality, Pärnu county	2.48 ± 0.05	5.57 ± 0.43	
3	Rosa glabrifolia	Suigu village, Are municipality, Pärnu county	4.19 ± 0.06	4.60 ± 0.14	
4	Rosa canina	Järvselja village, Meeksi municipality, Tartu county	2.50 ± 0.10	3.70 ± 0.29	
5	Rosa rugosavar.“Alba”	Alu township, Rapla municipality, Rapla county	Järvselja village, Meeksi municipality, Tartu county	0.67 ± 0.03	1.91 ± 0.11
6	Rosa nitida	Õssu village, Kambja municipality, Tartu county		1.65 ± 0.04	1.31 ± 0.08
7	Rosa vosagiaca	nc	Veeriku village, Valjala municipality, Saare county	nc	2.26 ± 0.17
8	Rosa mollis	nc		nc	1.28 ± 0.23
9	Rosa coriifolia	Kaisma village, Põhja-Pärnumaa municipality, Pärnu county		0.78 ± 0.03	1.32 ± 0.08
10	Rosa rugosavar.“Rubra”	nc	Rapla town, Rapla county	nc	1.22 ± 0.04
11	Rosa rubifolia		Järvselja village, Meeksi municipality, Tartu county	nc	4.41 ± 0.19
12	Rosa rugosa var. “Rosea”	Järvselja village, Meeksi municipality, Tartu county	nc	2.33 ± 0.06	nc
13	Rosa rugosa (violet)	Alu township, Rapla municipality, Rapla county	nc	1.87 ± 0.05	nc
14	Rosa gorenkensis	nc	Tallinn city	nc	4.71 ± 0.16

a nc–not collected. Only nine species were included in the redox assays due to the limited availability of sufficient plant material.

### 2.2 Effects of Rosehip Fruit Position on the Bush and its Growing Altitude on Ascorbic Acid Content

The *Rosa vosagiaca* fruits were from Tartu (Raja 31 Street, Tartu, Estonia), and *R. rugosa* from Uuri village (Kuusalu Rural Municipality, Harju County) were employed. In both cases, fruits were collected from different parts of one bush, according to latitude: north, east, south, and west, and from different heights in the southern part of the same bush: bottom, middle, and top. Rosehip picking took place in both cases at the end of October 2015. The collected fruits were immediately transferred to a freezer at −18 °C. The extraction of AA from fresh fruits and the HPLC analysis continued as described in the Supporting Information, Methods S2 and S3.

### 2.3 Effect of Rosehip Drying and Freezing on Ascorbic Acid Content

*Rosa rugosa* var. “*Rubra*” fruits, collected in Stockholm, Sweden, were used for drying experiments. For additional drying experiments at 40–80 °C and at 80 °C, *R. vosagiaca* fruits were used, collected from Tartu county. For drying experiments at temperatures of 20–120 °C were used *R. rugosa* fruits, collected from Harju County, Kuusalu Rural Municipality, Uuri village (Table 2). After collection, the fruits were transported to a freezer at −18 °C as soon as possible.

**Table 2:** Influence of the Drying Temperature (20-120 °C) to Ascorbic Acid Content

drying temperature, °C	content of ascorbic acid, % (dw)^a^
20	0.06 ± 0.00
40	0.10 ± 0.01
60	0.32 ± 0.03
80	0.38 ± 0.02
100	0.24 ± 0.01
120	0.15 ± 0.01

a Results are given as the mean of four replicates with the SD.

The fruits of three rosehip species (*R. pimpinellifolia* from Rapla town, Rapla municipality, Rapla district; *R. glabrifolia* from Suigu village, Are municipality, Pärnu district, and *R. mollis* from Veeriku village, Valjala municipality, Saare district, all in Estonia) were used for the investigation of the effect of freezing on AA content. Half of the fruits were stored in the freezer at −18 °C for a year, while the second half was analyzed immediately after drying at 80 °C for 5 h. Extraction of AA and HPLC analysis continued as described in Supporting Information S1a and S2.

### 2.4 Ascorbic acid Content (%) in Commercial Rosehips

Fifteen commercial rosehip preparations were collected: 10 from Estonia, 4 from Russia, and 1 from Lithuania. The preparations were purchased from both pharmacies and health food stores. The preparations were in different forms: dried whole rosehips (producers: MK Loodusravi, Fitofarm, Kubja Ürditalu, Vadi, EstVita, Health), crushed dried rosehips (Energia Talu, SVF), and crushed dried rosehips, which were in turn packaged in tea bags (Fitofarm, Krasnogorskleksredstva). Extraction of AA and HPLC analysis continued as described in Supporting Information S1a and S2.

### 2.5 Impact of Different Extraction Methods on Ascorbic Acid Yield

The aim of the extraction experiments was to determine which simple method of extraction allows the highest amount of AA to be isolated from rosehip drug under home conditions. A mixture of three commercial samples obtained from a community pharmacy was used to study the extraction methods (Vadi 2012, Vadi 2013, Uuskaubi Farm 2013). The corresponding methodology is described in Supporting Information (Methods S2 and S4).

### 2.6 Copper and Iron Reducing Capacity of Selected *Rosa* Samples

To complement the quantitative determination of AA and to assess its functional relevance, the copper- and iron-reducing capacities of selected rosehip samples were evaluated. The reducing activities were determined using established bathocuproine (BCS) and ferrozine assays. Methanolic extracts of rosehip fruits were prepared and tested at multiple concentrations under four (patho)­physiologically relevant pH conditions (4.5, 5.5, 6.8, and 7.5) using acetate or HEPES buffers. Reduction of cupric and ferric ions to their reduced forms was quantified spectrophotometrically in 96-well microplates by monitoring the formation of BCS–Cu­(I) and ferrozine–Fe­(II) complexes. A detailed description of reagents, experimental conditions, and analytical procedures is provided in the Supporting Information (Method S4).

### 2.7 Statistical Analysis

Basic parametric statistics were used to analyze the results. Data are expressed as mean values ± standard deviation (SD), typically based on four replicates. The mean values were compared using Student’s *t*-test for independent samples (two-tailed, assuming equal variances) when two groups were compared. Comparisons involving more than two groups were performed using one-way or two-way analysis of variance (ANOVA), as appropriate. Differences were considered statistically significant at *p* < 0.05. All statistical analyses were performed using GraphPad Prism software version 10.1.2 for Windows (GraphPad Software, USA). To compare the effects of the tested samples at different concentrations in the copper and iron reduction assays, reducing activities were analyzed using linear regression, and 95% confidence intervals were generated for the corresponding reduction curves.

## 3 Results

### 3.1 Ascorbic acid Content (%) in Rosehips from Different *Rosa* spp

The AA content varied markedly (*p* < 0.001) among the six *Rosa* species (Table 1, lines 1–6) and also differed between the two harvest years in a species-dependent manner (*p* < 0.05). The highest AA contents were observed in the fruits of *R. majalis* (5.57%), *R. gorenkensis* (4.71%), *R. glabrifolia* (4.60%), and *R. rubifolia* (4.41%). The AA content of rose hips collected in 2015 ranged from 0.26 to 4.19%, a difference of more than 16 times between the minimum and maximum. In 2023, the difference was even higher, with a range of 0.31 to 5.57%, an approximate 18-fold difference. The most common wild rose species in Estonia had different contents. Whereas *R. canina* had an average or high level (2.50–3.70%), the fruits of *R. rugosa* contained low to average amounts (0.67–2.33%).

### 3.2 Effects of Rosehip Location on the Bush on Ascorbic Acid Content

A statistically significant difference in AA content was observed between rosehip samples from Tartu city and Harju county (*p* < 0.05), regardless of fruit position on the bush (Figure 1). In both study locations, cardinal direction significantly influenced AA content, with consistently higher AA levels in west- and south-facing fruits compared to north-facing ones (*p* < 0.05), indicating higher AA accumulation in fruits exposed to greater solar radiation. In both study locations, bottom-position fruits consistently showed an AA content lower than that of middle and top fruits, which did not differ significantly from each other (*p* < 0.05).

**Figure 1:**
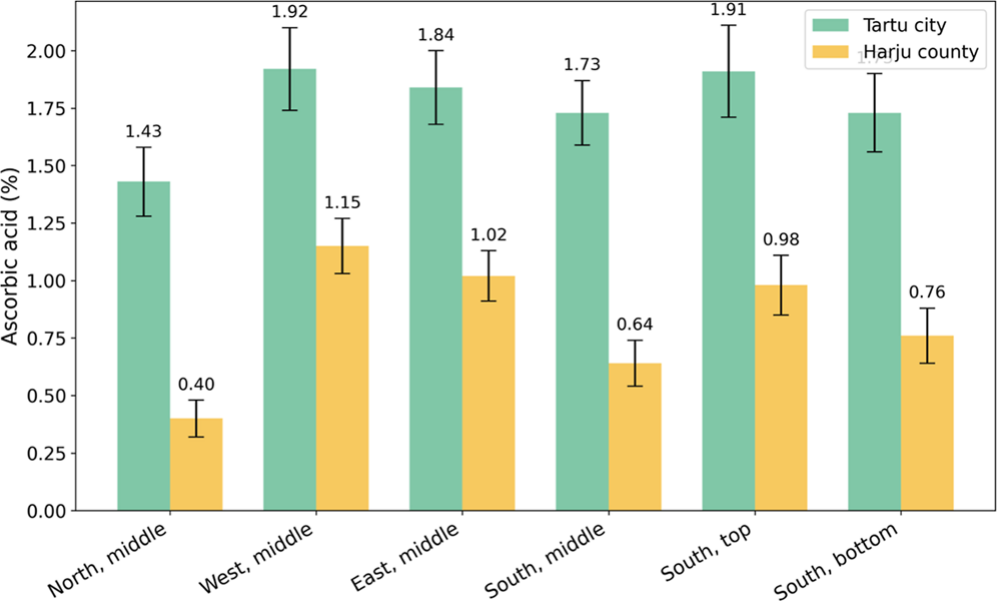
Ascorbic acid content (%) in rosehips depending on the location on the bush (north, south, east, and west) and height (top, middle, and bottom) compared to Tartu results with other results and similarly Harju results.

### 3.3 Effect of Drying and Freezing on Ascorbic Acid Content

The highest AA content in rose hips was achieved by freezing fresh fruits without drying (Figure 2A). Drying fruits at low (20–40 °C) or high temperatures for a long time (80 °C for 4 h) led to a decrease in AA content, while drying at 100 °C led to the decomposition of AA in the fruits within a few hours (*p* < 0.001), which was accompanied by the fruits turning black. Interestingly, the highest AA content was observed at 80 °C (*p* < 0.001), indicating that both lower and higher temperatures were associated with more rapid AA decomposition. Drying rosehip fruits at 80 °C for 6 h provides results comparable to drying at 60 °C for 1 day, while reducing the processing time by approximately 5-fold (Figure 2B). The content of AA was highest after 5–10 h of drying at 80 °C and declined significantly with longer drying times (Figure 2C; *p* < 0.001). Although, as above-reported, the AA content differed significantly among different rosehip samples, no significant differences were found between immediately dried and one-year frozen samples within the same species in all three selected fruit samples (*p* > 0.05, Figure 3).

**Figure 2:**
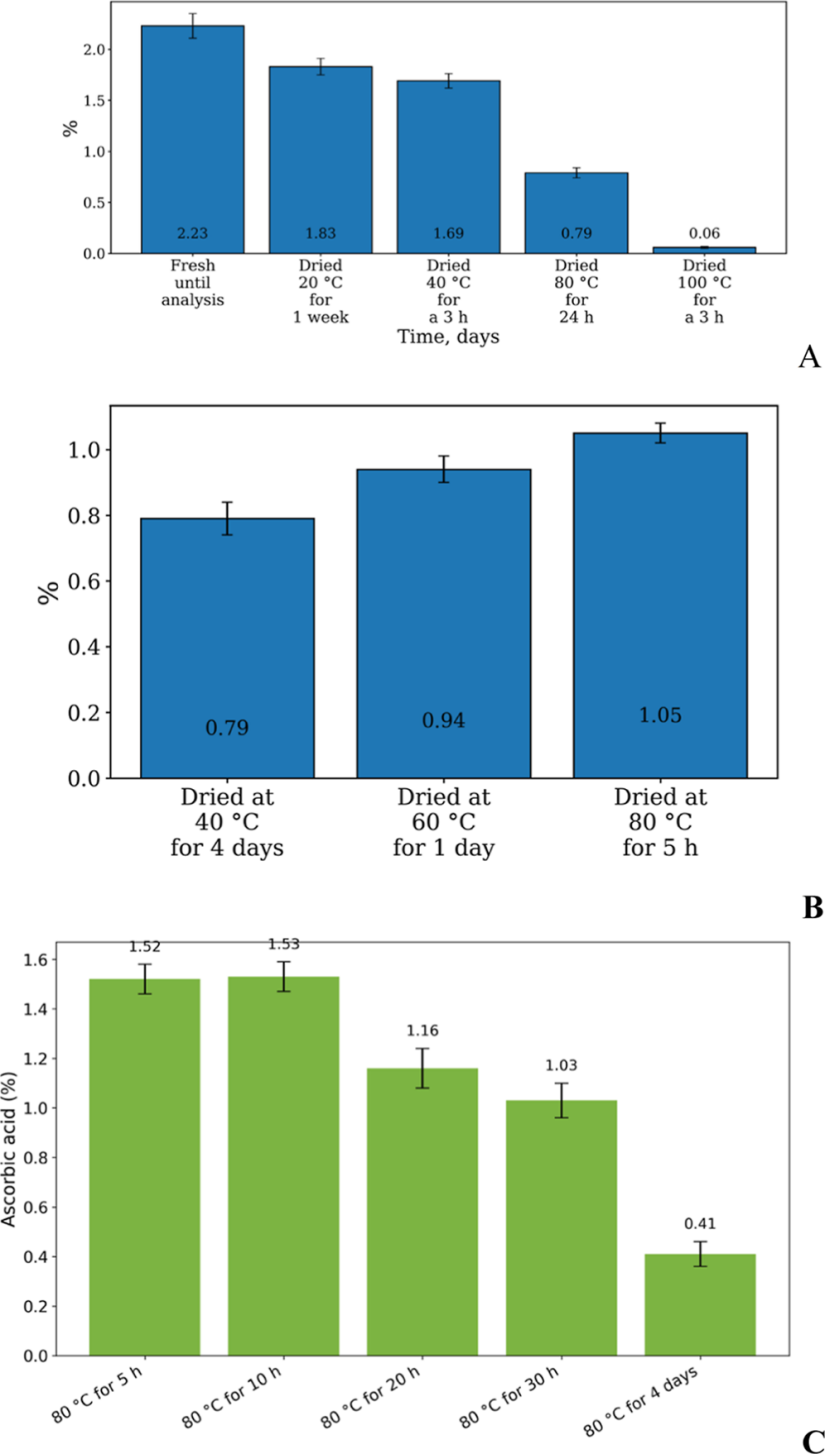
Impact of different methods of drying to the content of ascorbic acid. (A) Influence of drying temperature and time (20–100 °C) and drying time to AA content; (B) influence of temperature (40–80 °C) and drying time on AA content; (C) influence of drying time on 80 °C to the content of AA. Results are given as the mean of four replicates with SD.

**Figure 3:**
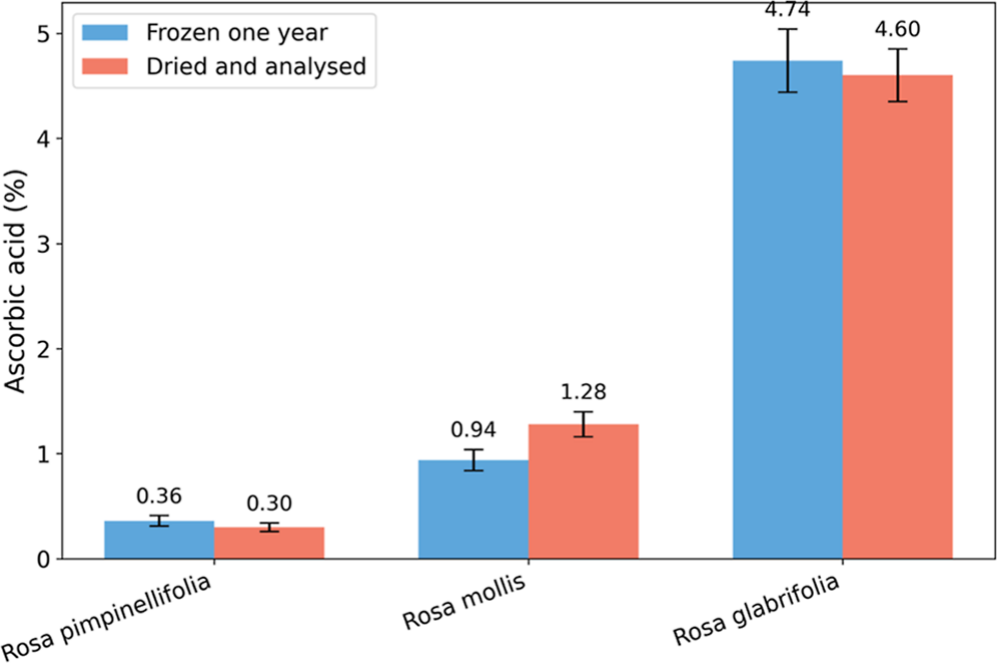
Content of ascorbic acid (%) in dried (80 °C) and frozen (−18 °C) rosehips. Results are given as the mean of four replicates with SD.

### 3.4 Ascorbic Acid Content (%) in Commercial Rosehips

A substantial proportion of commercial rosehip samples (nearly half of all tested products) contained no detectable AA (Table 3). In general, the AA content of commercial rose hips varies widely: if we exclude samples that do not contain AA, then the difference between the minimum and maximum content was 28-fold. Significant differences in AA content were observed between different production batches from the same manufacturer (*p* < 0.05), indicating pronounced batch-to-batch variability in commercial rosehip products. No consistent relationship between packaging form (tea bags with powdered and nonpowdered fruits) and AA content was observed (*p* > 0.05), suggesting that AA retention is primarily influenced by processing and storage conditions rather than packaging alone.

**Table 3:** Content of Ascorbic Acid (%) in Commercial *Rosa* spp. Fruits Available in the Community Pharmacies and/or in Health Food Stores

sample origin, package	content of AA, % (dw)
Estonia 1, 40 g[Table-fn t3fn1]	0.56 ± 0.03
Estonia 2, 40 g[Table-fn t3fn1]	0.38 ± 0.02
Estonia 3, 60 g	0.38 ± 0.03
Estonia 4, 50 g	0.16 ± 0.02
Estonia 5, 70 g[Table-fn t3fn1]	0.09 ± 0.00
Estonia 6, 70 g[Table-fn t3fn1]	0.05 ± 0.01
Estonia 7, 100 g[Table-fn t3fn1]	0
Estonia 8, 100 g[Table-fn t3fn1]	0
Estonia 9, 100 g[Table-fn t3fn1]	0
Estonia 10, 40 g[Table-fn t3fn1]	0
Lithuania 1, 100 g	0
Russia 1, tea bags 2 g × 20	0.36 ± 0.02
Russia 2, 100 g	0.02 ± 0.01
Russia 3, 100 g	0
Russia 4, tea bags 2 g × 20	0

a–d The same producer with different batch numbers.

### 3.5 Comparison of Home-Affordable Rosehip Extraction Methods Based on Ascorbic Acid Content

The highest AA extraction was achieved by boiling rosehips in water for 15 min, which has practical significance for preparing a vitamin C-rich infusion at home (Figure 4).

**Figure 4:**
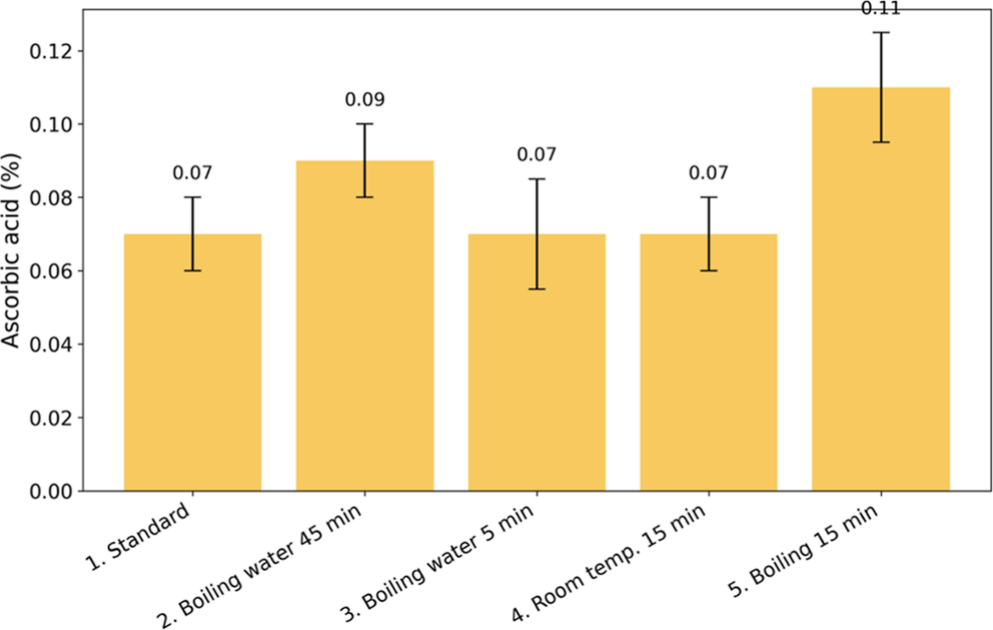
Extraction of ascorbic acid from dried and powdered rosehips.

1—reference extraction method (1% citric acid solution, stirred for 10 min); 2—infusion (boiling water, 45 min); 3—infusion (boiling water, 5 min); 4—maceration (room temperature water, 15 min); and 5—decoction (boiling for 15 min). Results are presented as the mean ± SD of four replicates.

The other four methods did not differ significantly from each other (*p* < 0.05). Neither shortening nor prolonging the extraction time following the addition of boiling water produced a significantly better effect on AA extraction. This observation suggests that the dense cellular structure typical of rosehip fruits may restrict the release of ascorbic acid during maceration, while sustained thermal treatment enhances the tissue disruption and compound diffusion.

### 3.6 Copper and Iron Reducing Capacity of Selected *Rosa* Samples

#### 3.6.1 Copper and Iron Reduction Assay

As the last step, we assessed the reducing properties of fruit samples from nine *Rosa* species toward cupric and ferric ions under four (patho)­physiologically relevant pH conditions. The reduction profiles varied between the samples (Figures 5, S1 and S2). In general, four types of reductive behavior were possible: (a) gradually linearly increasing, (b) bell-shaped, i.e., a decrease was observed after the rise, (c) absent, or (d) opposite, i.e., spontaneous reduction was inhibited (see Figures S1 and S2 for representative examples).

**Figure 5:**
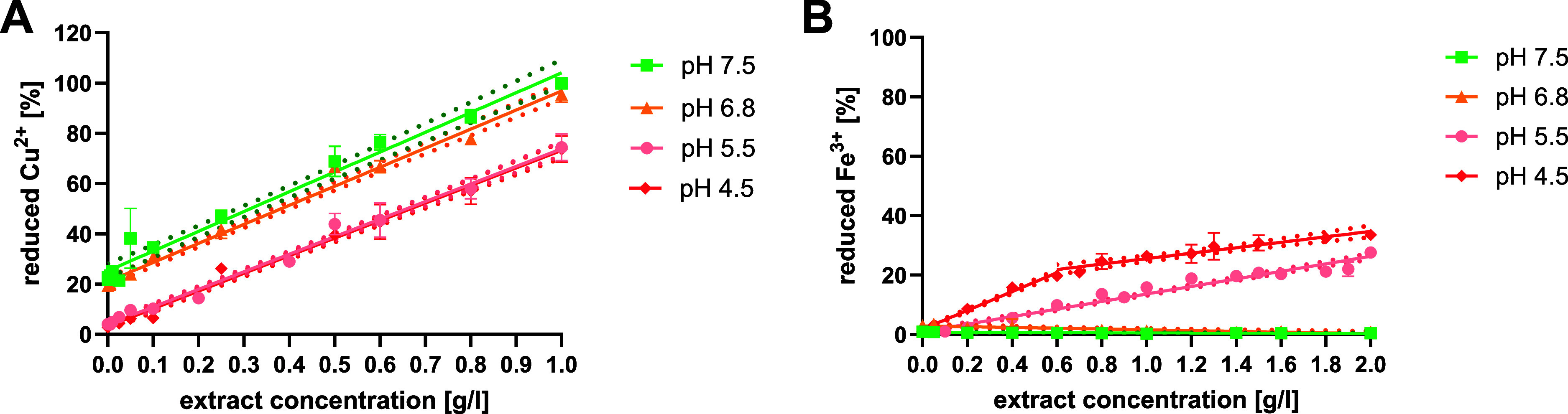
Representative example of the reduction activity of the *Rosa rugosa* var. *Rosea* tested by (A) BCS method and (B) ferrozine method at different pH values. The figure shows the measurement after 5 min. No ferric reduction activity was observed at pH 7.5 and 6.8.

All tested *Rosa* fruits were able to reduce cupric ions, with most of them achieving complete (100%) reduction after 5 min. Among the nine evaluated samples, only three (*R. rugosa* var.*alba*, *R. nitida*, and *R. Canina*) achieved complete reduction at all tested pH values. The reducing efficiency of the remaining compounds was pH-dependent. *R. pimpinellifolia* also achieved complete copper reduction but only at pH 5.5 and 4.5. *R glabrifolia* reached complete copper reduction as well but solely at pH 7.5 and 4.5. *R majalis* showed rather mild reducing effects at all of the pH levels. *R. rugosa* var. *Rubra*, and *R. coriifolia* also showed mild progressive reducing effects, but only at pH 6.8 and 7.5, with only minor effects observed at pH 5.5 and 4.5 (Figure S1).

In the case of ferric ion reduction, none of the tested species exhibited measurable activity at pH 6.8 or 7.5, whereas all species showed some degree of reduction under acidic conditions (pH 4.5 and 5.5) with markedly stronger effects at pH 4.5. However, the extent and pattern of reduction differed among the species. For example, *R. nitida* and *R. pimpinellifolia* displayed nonlinear behavior with decreased reduction at higher concentrations, whereas other species, such as *R. rugosa* var. *alba* and *R. canina*, showed more consistent, albeit moderate, reduction profiles. Overall, ferric ion reduction was weaker and more variable compared to copper reduction, indicating species-specific differences in matrix-dependent redox behavior.

When all species were included, no clear correlation between iron reduction and AA content was observed. However, *R. pimpinellifolia* deviated markedly from the general trend and was, therefore, considered a potential outlier. After exclusion of this species, a significant correlation emerged at pH 4.5 (*R* ^2^ = 0.72, *p* = 0.008; Figure S5). Both analyses (with and without the outlier) were presented to ensure transparency. The deviation of *R. pimpinellifolia* likely reflects species-specific matrix effects rather than analytical variability.

Subsequently, the reducing capacities of all tested compounds were compared using linear regression lines with 95% confidence intervals (Figures 6 and 7). The patterns of copper ion reduction observed at pH 5.5 and 4.5 were largely consistent across the compounds, indicating similar efficiency under mildly acidic conditions, while the pattern was dissimilar at higher pH levels. To facilitate comparison of the copper- and iron-reducing capacities across species and pH conditions, a schematic summary of relative reduction potentials is provided (Figure S3).

**Figure 6:**
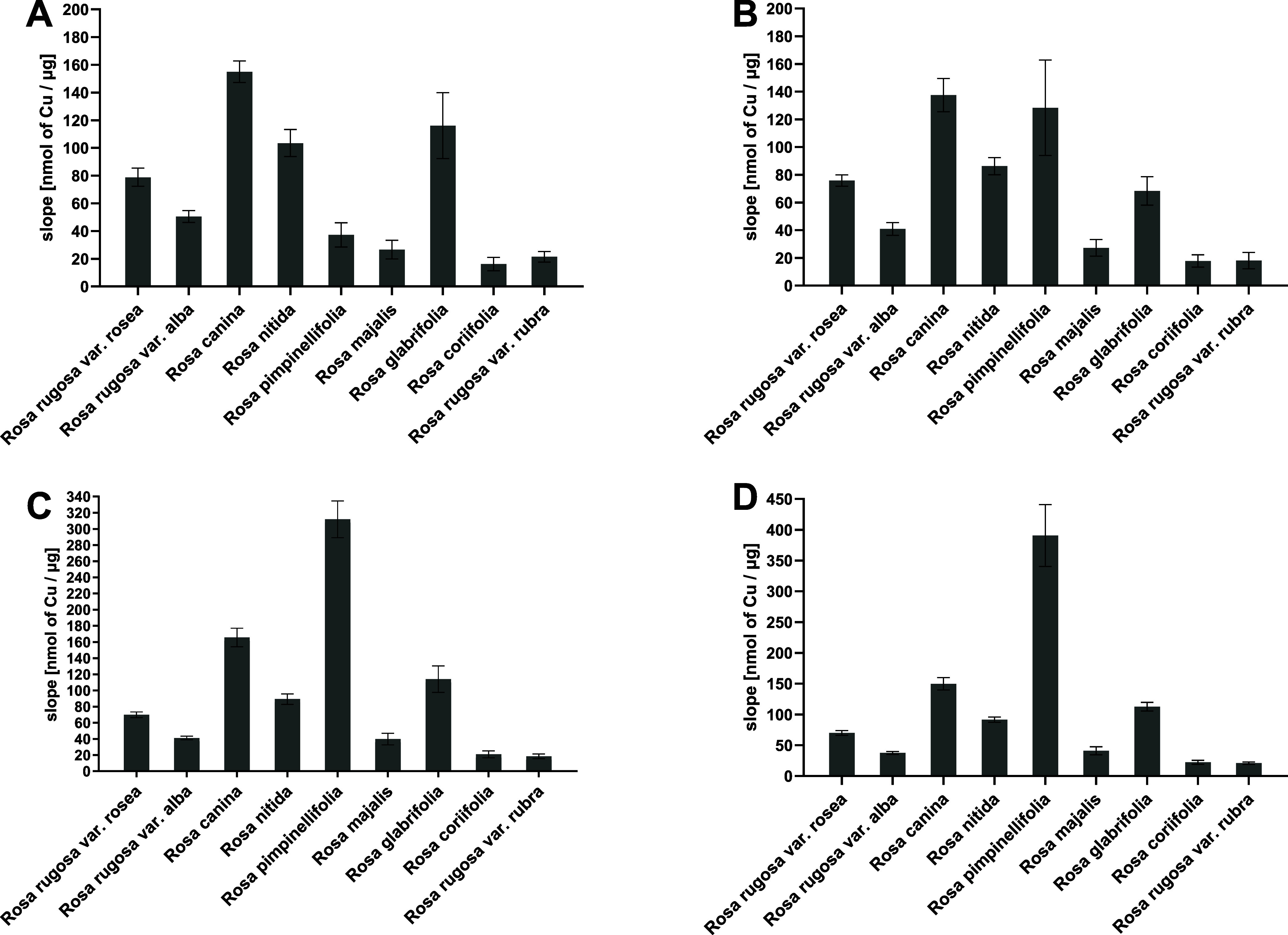
Copper reduction potency calculated from the slope of reduction lines at (A) pH 7.5, (B) pH 6.8, (C) pH 5.5, and (D) pH 4.5. Copper-reducing activity was assessed by constructing linear regression lines with 95% confidence intervals for the increase in copper reduction. The data represent measurements after 5 min.

**Figure 7:**
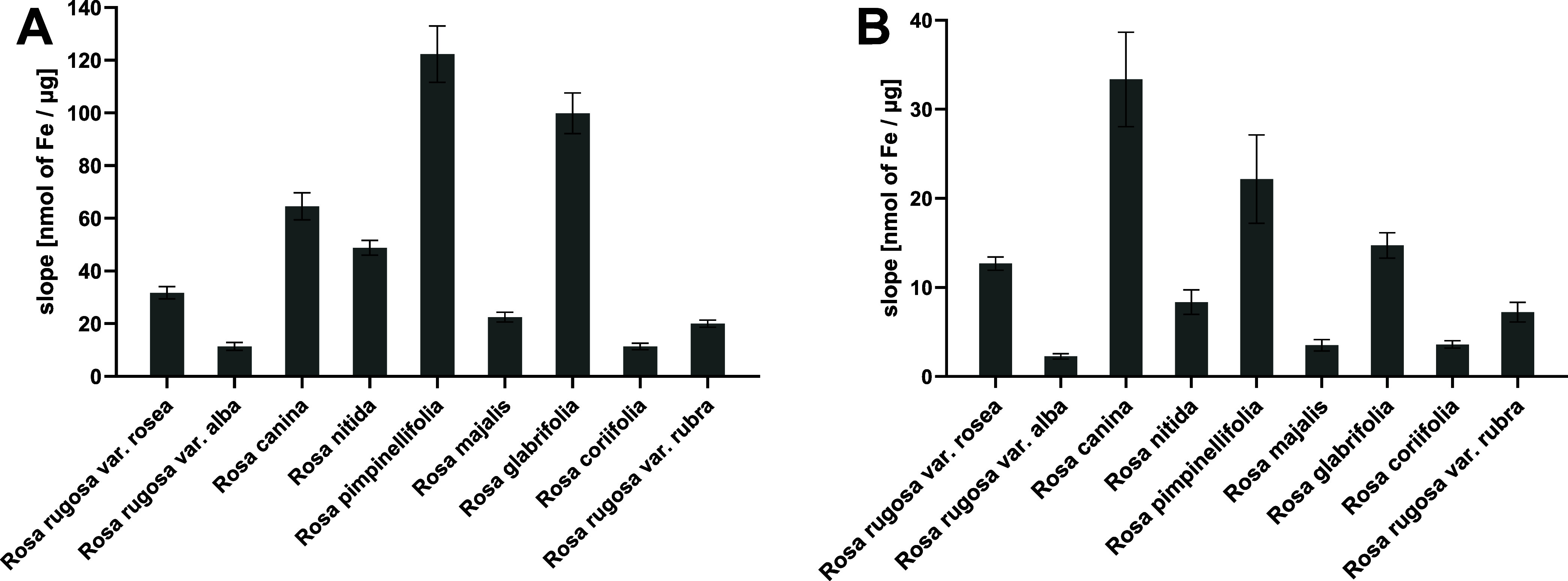
Iron reduction potency calculated from the slope of reduction lines at (A) pH 4.5 and (B) pH 5.5. Iron-reducing activity was assessed by constructing linear regression lines with 95% confidence intervals for the increase in iron reduction. The data represent measurements after 5 min.

No statistically meaningful correlation between copper reduction and AA content was observed, as indicated by the absence of a significant linear relationship (Figure S4).

## 4 Discussion

The present study focuses on AA as a quantitative marker, while other redox-active phytochemicals (e.g., polyphenols) were not individually characterized. Their contribution is therefore interpreted indirectly through functional redox assays. Our research shows that variability in AA content among *Rosa* fruits is accompanied by marked differences in functional redox behavior toward iron and copper. While quantitative differences in AA are strongly species- and processing-dependent, the reduction of metal ions is not governed by the concentration alone. In particular, the absence of correlation between copper reduction and AA content, together with pH-dependent, nonlinear iron reduction, indicates that species-specific phytochemical matrix effects play a decisive role. These findings place AA within a broader functional phytochemical context rather than treating it as an isolated antioxidant marker.

Ascorbic acid content in rosehip fruits has been widely investigated across different Rosa species, genotypes, geographic regions, and processing conditions.^5,6,23–25^ This study consistently confirms that rosehips are among the richest natural sources of vitamin C, while also demonstrating substantial variability in reported AA values. Such variability has been attributed to species- and genotype-specific differences, environmental and climatic factors, harvest year, postharvest processing, and analytical methodology.^6,23,24,26^ Substantial variation in vitamin C content has also been observed at the genotype level within rosehip species, further supporting the importance of genetic factors in determining AA accumulation.^27,28^ The AA content, depending on the altitude of growth, has also been shown.^26,29^

In addition to biological variability, postharvest handling represents another major source of variation in AA content. Contrary to the common assumption that AA is universally highly heat-labile, its stability depends strongly on oxygen availability, pH, and the surrounding matrix rather than on temperature alone.^30–32^ While thermal degradation may occur, degradation kinetics are largely governed by oxidative conditions and solution chemistry, particularly in aqueous or low-oxygen systems.^30,31^

The present results confirm that AA content in rosehips is strongly species-dependent, with highly significant differences among the investigated Rosa species, consistent with previous reports for the genus.^3,6,8,9,23,24,27^ Previous studies have consistently demonstrated that the AA content in rose hips is highly variable and strongly influenced by species and genotype. For example, high AA levels have been reported for commonly used species such as R. canina, *R. sempervirens*, and R. dumalis, with values ranging from approximately 180 to over 900 mg/100 g fresh weight, depending on genotype, maturity, and analytical approach.^7,33,34^ Similarly, elevated AA concentrations have been documented in less frequently studied taxa, including *R. sweginzowii*, *R. villosa*, and several Romanian genotypes, in some cases exceeding those of traditionally exploited rosehips.^6,35,36^ Previously, it was also reported that the highest vitamin C content was found in hip extracts of *R. rubiginosa* and *R. rugosa*.^25,27^ In line with this literature, our results show that the highest AA levels were recorded in *R. majalis*, *R. glabrifolia*, *R. rubifolia*, and *R. gorenkensis*, supporting the view that underutilized *Rosa* species may equal or even surpass widely used rosehips in vitamin C content.

Differences in light exposure and microclimatic conditions within plant canopies are known to influence the synthesis and accumulation of antioxidant compounds, including AA, because light intensity, quality, and associated temperature and water gradients affect carbon allocation and oxidative metabolism in plant organs.^30,37^ In rosehip fruits, greater light exposure within the bush is likely to promote higher AA accumulation, highlighting the role of microenvironmental conditions in shaping intraplant variability. The observed enrichment of AA in west- and south-facing fruits suggests that microclimatic gradients within the canopy represent an additional, previously overlooked source of variability in the rosehip vitamin C content. Although intraplant variation was observed, this factor was not further considered in the interpretation of redox behavior.

Our finding that drying temperature and duration markedly influence AA retention, with best retention at relatively high but brief drying (80 °C, 5–10 h) and substantial losses during prolonged drying and storage, agrees with previous investigations of rosehip processing and AA degradation kinetics (studies optimizing drying temperatures and modeling AA loss).^10,11^ The observed optimal retention of AA at 80 °C may reflect a balance between rapid moisture removal and limited oxidative degradation, as shorter drying times at moderately elevated temperatures can reduce exposure to oxygen and enzymatic activity. Freezing or low-temperature preservation has been reported to better maintain AA than extended hot-air drying,^38^ consistent with our −18 °C results.

Finally, copper and iron reduction assays complemented AA quantification by providing a functional measure of the redox activity. Copper-reducing capacity correlated with AA content across *Rosa* species (Figure S4), whereas iron reduction was weaker and strongly pH-dependent, indicating that probably other compounds modulate ferric ion reduction beyond AA concentration alone.^39,40^ Differences in both copper and iron reduction among species may be partly explained by variation in polyphenolic composition, as rosehip fruits contain flavonoids and other phenolic compounds with redox-active and metal-chelating properties; however, the partial correlation with ascorbic acid in the case of iron suggests a more complex interplay between AA and matrix components.^1^

This information is particularly important, as vitamin C is frequently used with ferrous ions in commercial tablets to increase the bioavailability of iron due to its reduction and/or stabilization in the ferrous form. Apparently, the effects of different *Rosa* fruits might be different, as the relationship is not linear in some tested species.^12^ This observation further supports the view that redox activity in plant extracts reflects the combined action of multiple phytochemical constituents rather than that of a single dominant compound. The nonlinear and pH-dependent iron reduction suggests competitive or antagonistic interactions within the phytochemical matrix.

While the observed lack of correlation between the AA content and copper reduction supports the conclusion that redox activity is not determined solely by AA, the interpretation in terms of phytochemical matrix effects remains indirect. The present study did not include compound-specific analysis of other redox-active constituents (e.g., polyphenols), and their contribution is therefore inferred rather than experimentally demonstrated. This represents a limitation of the study and should be addressed in future works.

Overall, these assays functionally corroborated the species-specific patterns revealed by AA quantification without introducing an independent ranking of the species. Taken together, these findings demonstrate that assessing vitamin-C-rich plant materials solely on the basis of AA concentration may overlook functionally relevant phytochemical interactions. The absence or very low levels of AA in a substantial proportion of commercial samples raise concerns regarding quality control, as vitamin C is widely used as a marker of rosehip product quality. These findings highlight the need for improved standardization and regulatory oversight to ensure the consistency and reliability of commercially available preparations.

## 5 Conclusions

This study demonstrates that AA content in rosehip (*Rosa* spp.) fruits is strongly dependent on species identity and postharvest processing, confirming pronounced variability within the genus. Short-term drying at moderate temperatures preserved AA most effectively, whereas prolonged drying led to substantial degradation, whereas freezing at – 18 °C maintained AA levels for 1 year. Functional redox assays showed that copper reduction is not correlated with AA content, whereas iron reduction is weaker, pH-dependent, and only partially associated with AA concentration. These findings indicate that redox behavior in rosehip fruits is not solely determined by AA concentration. The novelty of this work lies in combining multispecies comparison with functional redox assays.

## Supplementary Material



## Data Availability

AI-assisted language editing was performed using ChatGPT (OpenAI, GPT-5.2). The tool was used exclusively for English language refinement.

## Notes

Extraction of ascorbic acid (AA) from dried rosehips; HPLC analysis of AA in rosehips; extraction of AA from fresh rosehips; comparison of extraction methods of AA in rosehip; and copper and iron reduction assay (PDF)
