# Supplementary material for: Species- and Processing-Dependent Variability of Ascorbic Acid in Fruits of 14 Rosa Species and its Redox Behavior toward Iron and Copper Ions
Source: ACS Omega. 2026 May 22;11(22):32460–8. doi: 10.1021/acsomega.6c00617 (PMC13261576; doi:10.1021/acsomega.6c00617)
Supplement: Supplementary file 1 [file ao6c00617_si_001.pdf]

SUPPLEMENTARY MATERIAL

Species- and processing-dependent variability of ascorbic acid in fruits of 14 *Rosa* species and its redox behaviour toward iron and copper ions

Ain Raal[1*], Andres Meos[1], Inga Ainsaar[1], Olga Movtšanjuk[1], Kreete-Lisett Remmelgas[1], Oleh Koshovyi[1], Zuzana Lomozová[2], Patrícia Harčárová[2], Přemysl Mladěnka[3]

[1]Institute of Pharmacy, Faculty of Pharmacy, University of Tartu, Tartu, Estonia
[2] Department of Pharmacognosy and Pharmaceutical Botany, Charles University, Faculty of Pharmacy in Hradec Králové, Hradec Králové, Czechia
[3] Department of Pharmacology and Toxicology, Charles University, Faculty of Pharmacy in Hradec Králové, Hradec Králové, Czechia

Method S1. Extraction of ascorbic acid (AA) from dried rosehips

Before drying, the fruits were cut into pieces and the seeds were separated. The fruit shells were dried at a temperature of 80 °C for 5 h. Then the fruits were crushed in a coffee grinder and sieved through a sieve with a hole diameter of 2 mm. Four parallel extracts were prepared and analyzed from each herbal material. 10 ml of 1% citric acid aqueous solution (Method S1a) or 1% citric acid aqueous solution and 3% acetonitrile (Method S1b) were added to 0.5 g of the plant material. It is known that the stability of AA is higher in the acidic medium[1]. Extraction took place 1) 10 minutes in an ultrasonic bath, and 2) 10 minutes with a magnetic stirrer at medium speed. The samples were centrifuged for 5 minutes at 11,000 rpm, and then filtered through filter paper and the filtrate was once more filtered through a membrane filter into HPLC vials. Two HPLC vial samples were obtained from each sample: one using the described method and the other with 0.4 M dithiothreitol 1/20 of the sample amount. This was followed by HPLC analysis (Method S2**)**. The loss of drying of rosehip fruits was determined using the European Pharmacopoeia method [2] and the AA content was calculated on an absolute dry basis.

Method S2. HPLC analysis of AA in rosehips

The content of AA was determined by slightly modified European Pharmacopoeia HPLC method for quantification of related substances in AA as API (Ph. Eur., 2022, Monograph 01/2011:0253) [2]. Aminocolumn 250 mm x 4.6 mm, stationary phase particle size 5 µm (Phenomenex Luna® NH2) was used. The mobile phase was a mixture of acetonitrile and 0.05M aqueous solution of potassium-dihydrogen phosphate 75:25 (V/V), flow rate 1.0 ml/min, column temperature 45°C, detection wavelength 260 nm, 10 µl of the sample was injected. An assay was performed on HPLC PerkinElmer Series 200 HPLC (PerkinElmer, Inc., Waltham, MA, USA).), with autosampler and photoarray UV-detector. The system was controlled, and data was collected and processed by TotalChrom 6.3.2 software.

To test the system suitability resolution between the peaks due to AA and impurity C in the chromatogram obtained with reference solution (at least 3.0), as well as the signal-to-noise ratio for the peak due to impurity C in the chromatogram obtained with reference solution (at least 20) were determined.

We used the L-ascorbic acid standard obtained from Finnish pharmaceuticals company Oriola (Espoo, Finland) as a pharmaceutical active substance fulfilling the requirements of European Pharmacopoeia 4th ed. (Ph. Eur., 2010, Monograph 01/2011:0253) [2].

Daily, immediately before the assay, a ~0.2 mg/ml reference solution was prepared with mobile phase. A calibration curve was constructed to test the linearity between the standard's concentration and the corresponding peak's area. The correlation coefficient between these parameters within the AA concentration range from 0.50 to 0.01 mg/ml was close to 1 (y = 0.00000x + 0.00103, R^2 = 0.99998, r=0.99999).

Method S3. **Extraction of AA from fresh rosehips**

Frozen fruits (-18 °C) were thawed at room temperature and were cut into small strips with scissors and the seeds were removed. Four parallels were prepared from each sample. One rosehip fruit was used to prepare each parallel. 1 g of the cut fruit was precisely weighed. A 1% w/w citric acid water solution was prepared. 1 g of the cut fruit was placed in a mortar and 10 ml of 1% citric acid solution was poured on top. Then the resulting mixture was rubbed in a mortar with a pestle for five minutes to extract AA into the solution. The resulting extract was poured into test tube and were centrifuged for 5 minutes at 11,000 rpm. Four parallels were prepared in this way. The centrifuged samples were filtered through a membrane filter into separate HPLC vials. After filtration, the AA content in the samples was determined using the HPLC method as described in Method S2. The loss of drying of rosehip fruits was determined using the European Pharmacopoeia [2] method and the AA content was calculated on an absolute dry basis.

Method S4. Comparison of extraction methods of AA in rosehip

All dryied fruits (80⁰C for 5 h) were crushed with a coffee grinder, sieved (hole diameter 2 mm) and mixed together, to obtain the average of three commercial samples. Four parallel experiments were performed, each employing 5 g of the mixed powder. 100 ml of solvent was added to the powder and five extraction methods were performed:

1. Reference extraction method**:** 1% (w/w) citric acid aqueous solution was poured onto the drug powder and stirred for 10 min on a magnetic stirrer.
2. Infusion (45 min)**:** boiling water was poured onto the drug powder and left to stand for 45 min.
3. Infusion (5 min): boiling water was poured onto the drug powder and left to stand for 5 min.
4. Maceration (15 min): room-temperature water was poured onto the drug powder and left to stand for 15 min.
5. Decoction (15 min): the drug was mixed with water and boiled for 15 min.

After extraction, the obtained extracts were filtered through a filter paper and then centrifuged for 5 minutes at a speed of 11,000 rpm. The centrifuged samples were again filtered through a membrane filter into separate HPLC vials and HPLC analysis was performed as described in Method S2.

Method S5. Copper and iron reduction assay

Reagents, solutions, and equipment for reduction experiments

Ferric chloride hexahydrate ($FeCl_3 \cdot 6H_2O$), cupric sulfate pentahydrate ($CuSO_4 \cdot 5H_2O$), disodium bathocuproine disulfonate (BCS), 3-(2-pyridyl)-5,6-diphenyl-1,2,4-triazine-4′,4′′-disulfonic acid sodium salt (ferrozine), hydroxylamine hydrochloride (HA), acetic acid, sodium acetate, 4-(2-hydroxyethyl)-1-piperazineethanesulfonic acid (HEPES), HEPES sodium salt were purchased from Sigma-Aldrich (Munich, Germany). Methanol was purchased from

Thermo Fisher Scientific (Pardubice, Czech Republic). Ultrapure water (Milli-Q RG, Merck Millipore, Burlington, MA, USA) was used throughout this study.
Stock solutions of cupric ions, ferric ions, ferrozine, and BCS (concentrations were 5 mM), and HA (100 mM) were prepared in ultrapure water. The tested rosehips samples were extracted using methanol to obtain final concentrations of 5 mg/mL or 2 mg/mL, put in an ultrasonic bath for 10 minutes, stirred with a magnetic stirrer at medium speed for 10 minutes, and filtered through a filter paper. Lower concentrations were prepared by dilution with methanol.

Copper and iron reduction experiments were performed at four (patho)physiologically relevant pH values (4.5, 5.5, 6.8, and 7.5). Acetate buffers (15 mM of sodium acetate with 27.3 and 2.7 mM of acetic acid, respectively) were used for the two lower pH values, whereas HEPES buffers (15 mM of sodium HEPES with 71.7 and 14.3 mM of HEPES, respectively) were used for pH 6.8 and 7.5.

Reduction experiments were performed in 96-well microplates (BRAND GmbH&Co. KG, Wertheim, Germany) with Hidex Sense Beta Plus microplate reader (Hidex, Turku, Finland).

Copper and iron reduction assay

The degree of copper and iron reduction was established by the use of BCS and ferrozine methodology as previously reported by us [4,5]. The principle of these methods is that oxidized cupric or ferric ions do not react with the indicator BCS/ferrozine, in contrast to reduced cuprous or ferrous ions. When a tested sample reduces cupric ions into cuprous ions or ferric ions into ferrous ions, the indicator rapidly forms a complex with them, which is thereafter measured spectrophotometrically. Absorbance was measured immediately after the addition of indicators and 5 min later. Every sample was tested at least in 6 different concentrations in order to construct concentration-dependent curves. All experiments were performed in 96-well microplates.

The ferrozine method

Ferrozine is a specific reagent that forms a magenta-colored complex with ferrous ions with an absorption maximum at λ =562 nm. Different concentrations of tested samples (50 µL) in methanol were mixed with a solution of ferric ions (50 µL, 250 µM) in acetate (pH 4.5 and 5.5) or HEPES buffers (pH 6.8 and 7.5) for 2 min at room temperature. Subsequently, an aqueous solution of ferrozine (50 µL, 5 mM) was pipetted, and the absorbance of the resulting purple complex was measured at λ 562 nm. HA served as the positive control, representing a 100% reduction [4].

The BCS method

The BCS method is analogous to the ferrozine method, but BCS is specific for cuprous ions. Various buffers were first mixed with different concentrations of the tested samples (50 µL) dissolved in methanol. A solution of cupric ions (50 µL, 250 µM) was added, and the mixture was stirred for 2 min at room temperature. Afterwards, an aqueous solution of BCS (50 µL, 5 mM) was added, and absorbance was measured at λ = 484 nm. Hydroxylamine was used as the positive control (100% copper reduction). A more detailed methodology can be found in our former study [5].

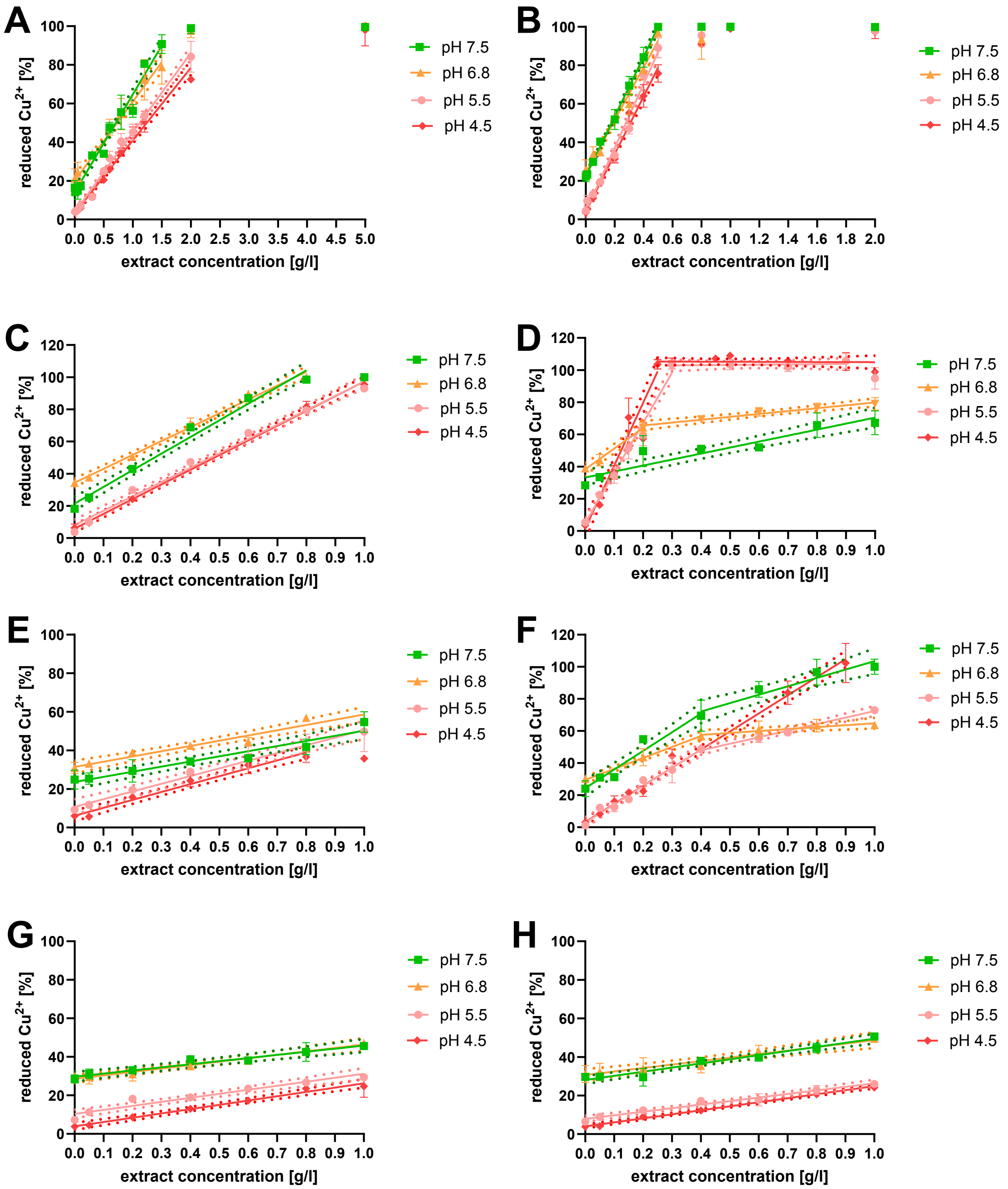

Figure. S1. Copper reduction tested by the bathocuproine method. **A**: *Rosa rugosa* var. *alba*, **B**: *Rosa canina*, **C**: *Rosa nitida*, **D**: *Rosa pimpinellifolia*, **E**: *Rosa majalis*, **F**: *Rosa glabrifolia*, **G**: *Rosa coriifolia*, **H**: *Rosa rugosa* var. *rubra*. Data are shown as mean ± SD and are from measurements after 5 min.

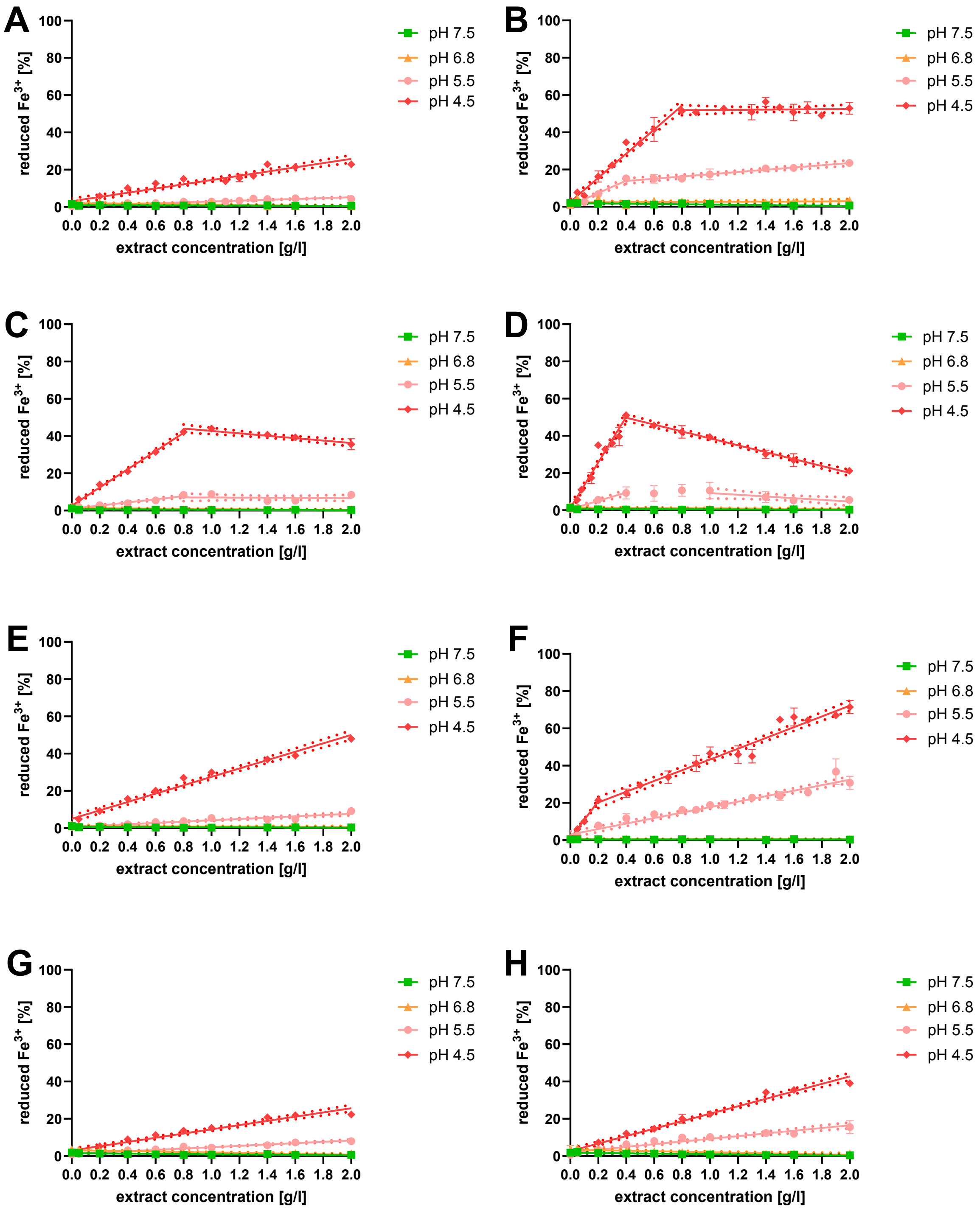

Figure. S2. Iron reduction tested by the ferrozine method. **A**: *Rosa rugosa* var. *alba*, **B**: *Rosa canina*, **C**: *Rosa nitida*, **D**: *Rosa pimpinellifolia*, **E**: *Rosa majalis*, **F**: *Rosa glabrifolia*, **G**: *Rosa coriifolia*, **H**: *Rosa rugosa* var. *rubra*. Data are shown as mean ± SD and are from measurements after 5 min.

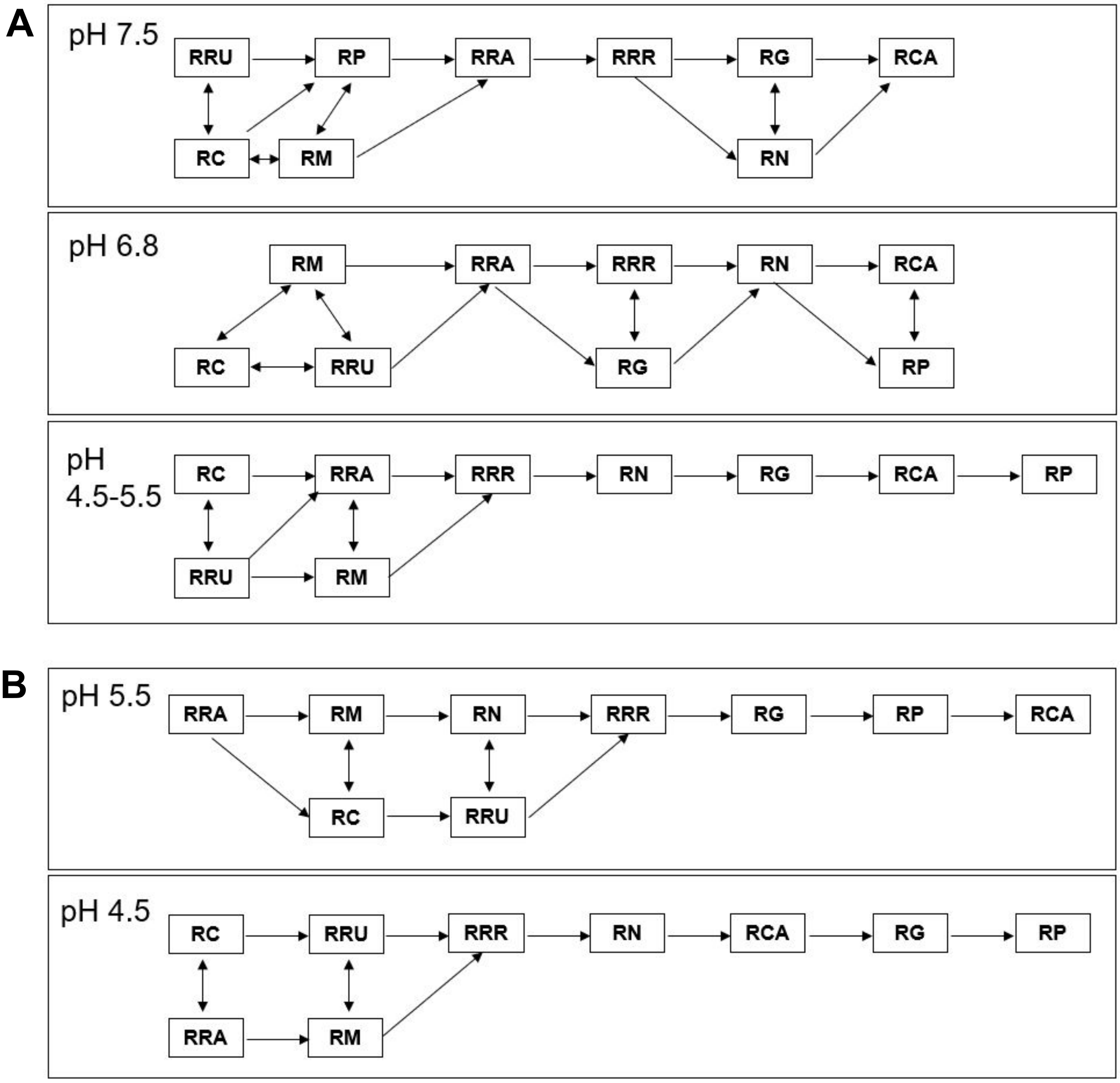

Figure S3: Schema showing differences in reduction potential of cupric and ferric ions at all tested pH levels. **A:** cupric reduction, **B:** ferric reduction. Direction of arrows shows a more powerful ferric or copper ions reducing agent, double-headed arrow means no significant difference.

RRR: *Rosa rugosa* var. *rosea*, **RM**: *Rosa majalis*, **RC**: *Rosa coriifolia*, **RRU**: *Rosa rugosa* var. *rubra*, **RN**: *Rosa nitida*, **RRA**: *Rosa rugosa* var. *alba*, **RG**: *Rosa glabrifolia*, **RP**: *Rosa pimpinellifolia*, **RCA**: *Rosa canina*

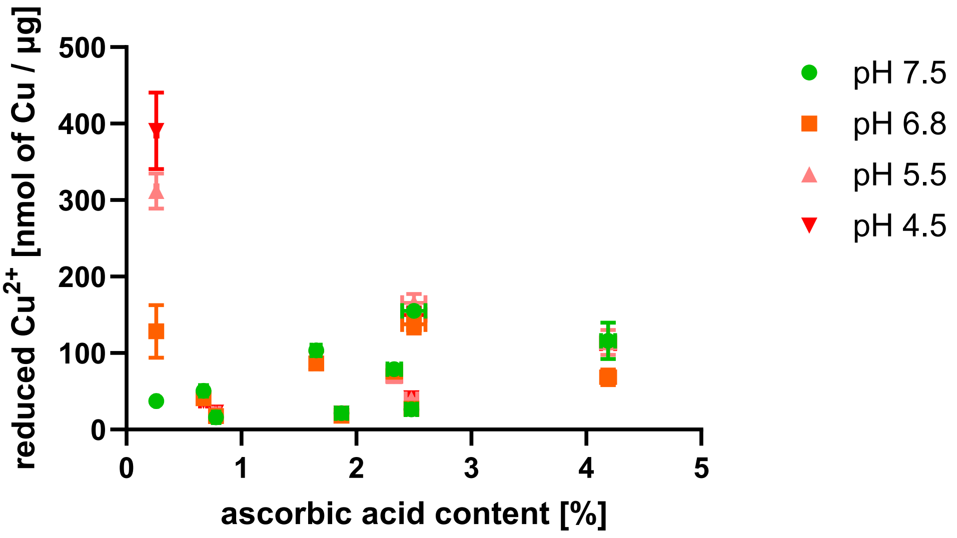

Figure S4. No correlation between copper reduction and vitamin C content was found.

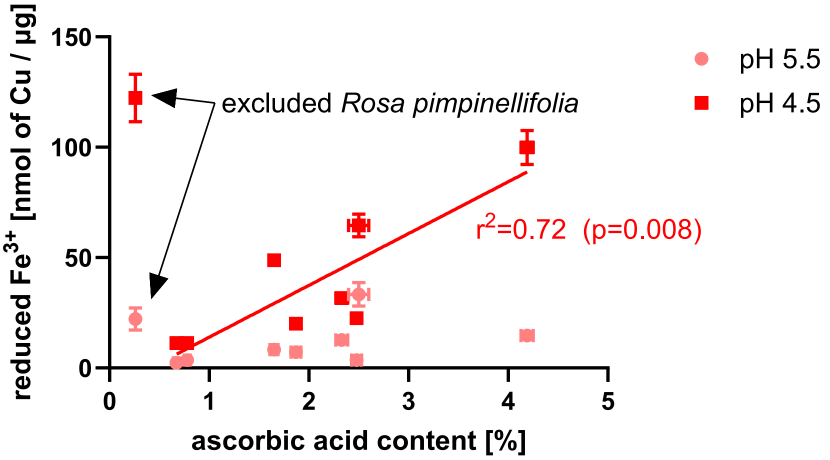

Figure S5: Correlation between iron reduction and vitamin C content. A correlation between ferric iron reduction and ascorbic acid content was found at pH 4.5 when apparently outlying *Rosa pimpinellifolia* species was removed from the analysis. No relationship was found at pH 5.5.

References

(1) Golubitskii, G. B.; Budko, E. V.; Basova, E. M.; Kostarnoi, A. V.; Ivanov, V. M. Stability of Ascorbic Acid in Aqueous and Aqueous-Organic Solutions for Quantitative Determination. *J Anal Chem* **2007**, *62* (8), 742–747. https://doi.org/10.1134/S1061934807080096.

(2) *European Pharmacopoeia*, 11th ed.; Council of Europe: Strasbourg, 2022.

(3) Meos, A.; Zaharova, I.; Kask, M.; Raal, A. Content of Ascorbic Acid in Common Cowslip (Primula Veris L.) Compared to Common Food Plants and Orange Juices. *Acta Biologica Cracoviensia s. Botanica* **2017**, *59* (1), 113–120. https://doi.org/10.1515/abcsb-2016-0020.

(4) Mladěnka, P.; Macáková, K.; Zatloukalová, L.; Řeháková, Z.; Singh, B. K.; Prasad, A. K.; Parmar, V. S.; Jahodář, L.; Hrdina, R.; Saso, L. In Vitro Interactions of Coumarins with Iron. *Biochimie* **2010**, *92* (9), 1108–1114. https://doi.org/10.1016/j.biochi.2010.03.025.

(5) Říha, M.; Karlíčková, J.; Filipský, T.; Macáková, K.; Hrdina, R.; Mladěnka, P. Novel Method for Rapid Copper Chelation Assessment Confirmed Low Affinity of D-Penicillamine for Copper in Comparison with Trientine and 8-Hydroxyquinolines. *Journal of Inorganic Biochemistry* **2013**, *123*, 80–87. https://doi.org/10.1016/j.jinorgbio.2013.02.011.
